# m^6^A modification-mediated BATF2 acts as a tumor suppressor in gastric cancer through inhibition of ERK signaling

**DOI:** 10.1186/s12943-020-01223-4

**Published:** 2020-07-10

**Authors:** Jian-Wei Xie, Xiao-Bo Huang, Qi-Yue Chen, Yu-Bin Ma, Ya-Jun Zhao, Li-Chao Liu, Jia-Bin Wang, Jian-Xian Lin, Jun Lu, Long-Long Cao, Mi Lin, Ru-Hong Tu, Chao-Hui Zheng, Chang-Ming Huang, Ping Li

**Affiliations:** 1 Department of Gastric Surgery, Fujian Medical University Union Hospital, No.29 Xinquan Road, Fuzhou, 350001 Fujian Province China; 2 Key Laboratory of Ministry of Education of Gastrointestinal Cancer, Fujian Medical University, Fuzhou, China; 3 Fujian Key Laboratory of Tumor Microbiology, Fujian Medical University, Fuzhou, China; 4 Department of Gastrointestinal Surgery, the Affiliated Hospital of Qinghai University, Xining, China; 5 Department of Gastrointestinal Surgery, the First Affiliated Hospital of USTC, Division of Life Sciences and Medicine, University of Science and Technology of China, Hefei, China

**Keywords:** Gastric cancer, BATF2, ERK, p53, m^6^A

## Abstract

**Background:**

BATF2, also known as SARI, has been implicated in tumor progression. However, its role, underlying mechanisms, and prognostic significance in human gastric cancer (GC) are elusive.

**Methods:**

We obtained GC tissues and corresponding normal tissues from 8 patients and identified BATF2 as a downregulated gene via RNA-seq. qRT-PCR and western blotting were applied to examine BATF2 levels in normal and GC tissues. The prognostic value of BATF2 was elucidated using tissue microarray and IHC analyses in two independent GC cohorts. The functional roles and mechanistic insights of BATF2 in GC growth and metastasis were evaluated in vitro and in vivo.

**Results:**

BATF2 expression was significantly decreased in GC tissues at both the mRNA and protein level. Multivariate Cox regression analysis revealed that BATF2 was an independent prognostic factor and effective predictor in patients with GC. Low BATF2 expression was remarkably associated with peritoneal recurrence after curative gastrectomy. Moreover, elevated BATF2 expression effectively suppressed GC growth and metastasis in vitro and in vivo. Mechanistically, BATF2 binds to p53 and enhances its protein stability, thereby inhibiting the phosphorylation of ERK. Tissue microarray results indicated that the prognostic value of BATF2 was dependent on ERK activity. In addition, the N6-methyladenosine (m^6^A) modification of BATF2 mRNA by METTL3 repressed its expression in GC.

**Conclusions:**

Collectively, our findings indicate the pivotal role of BATF2 in GC and highlight the regulatory function of the METTL3/BATF2/p53/ERK axis in modulating GC progression, which provides potential prognostic and therapeutic targets for GC treatment.

## Background

Although radical resection and systemic chemotherapy have shown great improvements, the prognosis of patients with gastric cancer (GC) is still dismal, due to malignant proliferation and metastasis [1]. Thus, elucidating the underlying molecular mechanisms of GC progression is urgently needed and will contribute to the development of targeted therapies. Extracellular signal-regulated kinase (ERK) signaling is frequently activated and participates in GC progression [2, 3]. Precise regulation of ERK activation is essential to convey appropriate signals [4]. Hence, it is of interest to explore the potential regulatory mechanisms of ERK signaling in GC.

The basic leucine zipper ATF-like transcription factor 2 (BATF2; also known as SARI) gene is located on human chromosome 11q and contains a basic leucine zipper protein domain, as do its family members BATF1 and BATF3 [5]. Initially, BATF family members were suggested to be AP-1 inhibitors [6], but in recent years, oncologists have indicated that BATF2 is also involved in the progression of cancers through diverse mechanisms. Wang et al. [7] demonstrated that loss of BATF2 significantly promotes tumor metastasis by initiating epithelial-mesenchymal transition (EMT) and induces apoptosis in lung adenocarcinoma. Furthermore, BATF2 attenuates breast cancer cell proliferation and invasion by suppressing the transcription of CCN1 and inhibiting the expression of c-Jun [8]. Moreover, a novel study showed that when BATF2 is induced, it inhibits angiogenesis by directly binding to ceruloplasmin, thereby blocking the HIF-1α/VEGF axis in colon cancer [9]. However, whether and how BATF2 regulates ERK signaling in GC remain elusive.

By investigating the upstream regulatory mechanisms of BATF2, we found that its mRNA level may be related to the N6-methyladenosine (m^6^A) methylation modification. m^6^A methylation is the most prevalent internal chemical modification in eukaryotic mRNAs [10]. Previous studies reported that the effects of m^6^A modification include regulating mRNA stability, splicing, and translation [11–13]. Methyltransferase-like 3 (METTL3) is a catalytic enzyme that promotes m^6^A modification of mRNAs [14]. However, the mechanism by which METTL3 mediates the m^6^A modification of BATF2 to affect GC progression has not been reported.

In the present study, we investigated the clinical significance and regulatory mechanisms of BATF2 in GC. We identified that BATF2 acts as a tumor suppressor in GC by inhibiting cell proliferation, migration, and invasion. We further proposed that METTL3/BATF2/p53/ERK signaling axis enriches current understanding of GC progression. This implies that BATF2 is a potential predictive biomarker and therapeutic target for GC.

## Methods

### Human gastric tumor tissues

Gastric tumor tissues from 843 patients were obtained from Fujian Medical University Union Hospital (Fuzhou, China), the Affiliated Hospital of Qinghai University (Xining, China) and the First Affiliated Hospital of USTC (Hefei, China). The internal cohort included 611 paired gastric tumor tissues and adjacent nontumor tissues from Fujian Medical University Union Hospital. The external validation cohort comprised 232 gastric tumor tissues and 50 adjacent nontumor tissues from the Affiliated Hospital of Qinghai University and the First Affiliated Hospital of USTC. The inclusion criteria were as follows: (a) histological identification of GC; (b) the absence of combined malignancy and distant metastasis; (c) availability of complete follow-up data; and (d) tumor stage classification carried out or updated according to the 7th edition of the AJCC cancer staging handbook [15]. Patients who died within 1 month or received prior chemotherapy or radiotherapy at the time of gastrectomy were excluded. For the internal cohort, 259 fresh gastric tumor tissues and adjacent nontumor tissues were collected randomly between 2017 and 2018; 124 samples were subjected to western blotting, and the other 135 were used for qRT-PCR analysis. Moreover, 584 paraffin-embedded gastric tumor tissues collected from 2010 to 2015 from the two independent cohorts were used for immunohistochemistry (IHC). Among 352 patients with GC from the internal cohort, 96 cases were tested for *H. pylori* infection. This study was approved by the ethics committee of Fujian Medical University Union Hospital (No. 2020KY042), and written consent was obtained from all enrolled patients.

### Follow-up

All patients were systematically followed up by trained doctors who abided by the institutional follow-up protocol; options for follow-up included outpatient services, letters, telephone, mail or visits. Follow-up was conducted every 3–6 months for the first 2 years, every 6–12 months for the 3–5 years, and annually thereafter. Survival time was defined as the time from the date of surgery until the date of last follow-up or death. All 584 patients involved in the IHC analysis completed the follow-up.

### Public datasets

The ACRG (GSE62254) and TCGA-STAD (The Cancer Genome Atlas Stomach Adenocarcinoma) datasets were analyzed in this study. ACRG dataset was available from the Gene Expression Omnibus (GEO) database (http://www.ncbi.nlm.nih.gov/geo/). Among 300 patients with GC from the ACRG dataset, 127 cases were tested for *H. pylori* infection (55 cases in *H. pylori*-positive group, 72 cases in *H. pylori*-negative group). TCGA-STAD dataset was available from the Genomic Data Commons (GDC) Data Portal (http://portal.gdc.cancer.gov/) using the GDC Data Transfer Tool.

### RNA-sequencing analysis

Total RNA extraction was performed with TRIzol Reagent (Invitrogen, Carlsbad, CA, USA). RNA-sequencing analysis was performed at KangChen Bio-tech Inc. (Shanghai, China).

### Tissue microarray (TMA)

A series of TMAs containing GC samples were constructed. Briefly, all GC tissues were reviewed by pathologists, and representative areas free from necrotic and hemorrhagic materials were marked in paraffin blocks. For each sample, a 1.5-mm core was punched from the donor blocks and transferred to the recipient paraffin block at defined array positions using a TMA instrument. Several serial sections (4 μm in thickness) were cut from all TMAs, and one section was stained with H&E (hematoxylin and eosin) as a reference.

### Establishment of cell lines

Overexpression and knockdown lentiviruses for BATF2 (NM_138456) as well as control lentivirus were purchased from GeneChem Corporation (Shanghai, China). Transfection was performed according to the manufacturer’s instructions. Puromycin (2 μg/ml, Sigma) was used to select stable clones for at least 1 week. At the indicated time points, the cells were harvested for mRNA and protein analysis as well as for other assays.

### Tumor formation and metastasis assays

All male BALB/c nude mice (4–5 weeks old) used in our study were purchased from Beijing Vital River Laboratory Animal Technology Co., Ltd. A total of 5 × 10^6^ stably transfected HGC-27 cells were subcutaneously injected into the right axillary fossa of nude mice. Tumor volume was measured every 3 days and calculated with the following formula: V = (L × W^2^)/2 cm^2^ (V, tumor volume; L, length; W, width). The mice were sacrificed at 3–4 weeks after injection, and the tumors were weighed. For the lung metastasis model, 5 × 10^6^ stably transfected HGC-27 cells were injected into the tail veins of nude mice. Forty-five days later, the mice were sacrificed, and the lungs were dissected to examine the histopathological metastatic loci. The peritoneal dissemination ability of GC cells was evaluated via intraperitoneal injection. A total of 5 × 10^6^ stably transfected HGC-27 cells in 500 μl of PBS were injected into the peritoneal cavity of BALB/c nude mice. Mice were carefully monitored until they were killed at 4 weeks, at which point peritoneal metastases were examined and recorded.

All animal experiments were performed according to the Animal Protection Committee of Fujian Medical University (Fuzhou, China) and approved by the Ethics Committee of Fujian Medical University/Laboratory Animal Center (Fuzhou, China).

### Proteome phospho-kinase array

The relative levels of phosphorylated protein were tested using the Proteome Profiler Human Phospho-Kinase Array Kit (ARY003B, R&D Systems, Inc. USA & Canada) according to the manufacturer’s protocol. Equal amounts of protein (600 mg) were extracted from stably transfected cells (BATF2-overexpressing HGC-27 cells and BATF2-knockdown SNU-216 cells), and the kinase activity of BATF2 was compared.

### RNA immunoprecipitation (RIP)

BATF2 m^6^A immunoprecipitation was performed using a Magna MeRIP m^6^A Kit (17–10499, Merck Millipore, USA) according to the manufacturer’s protocol, and the immunoprecipitated RNA extracts were reverse-transcribed and analyzed by qRT-PCR.

### Dual-luciferase reporter assay

The luciferase reporter assay was performed with the Dual-Luciferase Reporter Assay System (Promega) according to the manufacturer’s protocol. Cells seeded in 24-well plates were transfected with pmiGLO-based luciferase vector fused or not fused to the wild-type or mutated BATF2–3′UTR. METTL3 or empty vectors were cotransfected. Firefly luciferase activity was normalized to Renilla luciferase activity to reflect transfection efficiency. Experiments were performed in triplicate.

### Statistical analysis

Statistical analysis was performed using SPSS software, version 16.0 (IBM Corporation, New York, USA). Data were presented as the mean ± SD and analyzed using Student’s t test or one-way ANOVA. The chi-squared test was applied to examine the association between BATF2 expression and clinicopathological parameters. Kaplan-Meier analysis was used to compare the survival distributions of the two groups with the log-rank test. Prognostic factors were examined by univariate and multivariate analyses using the Cox proportional hazards model. Spearman’s rank analysis was performed to determine the correlation between different protein levels. *P* < 0.05 was considered statistically significant.

Other details and additional experimental procedures are provided in Additional file 1: Supplementary Materials and Methods.

## Results

### Decreased BATF2 expression correlates with poor prognosis of patients with GC

**Fig. 1:**
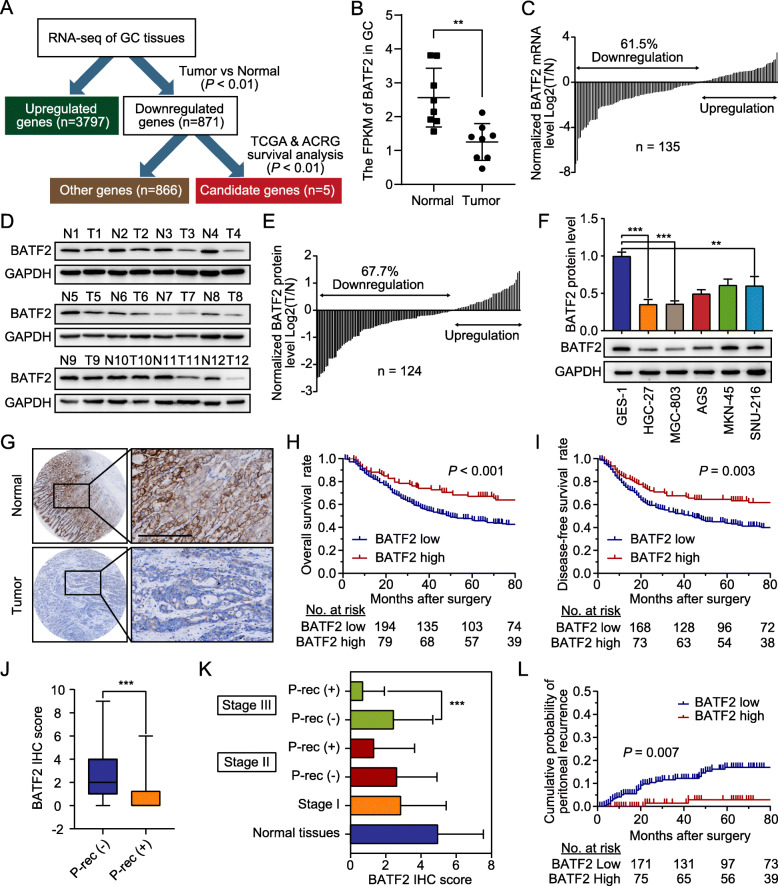
The expression and prognostic value of BATF2 in GC. **a** Flowchart of the screening process of candidate genes. **b** The FPKM (fragments per kilobase million) of BATF2 in gastric tumor and adjacent normal tissues. **c** The mRNA levels of BATF2 in gastric tumor and adjacent normal tissues were measured by qRT-PCR. **d** Representative images of BATF2 protein levels in gastric tumor and adjacent normal tissues. **e** The T/N ratios of the total results described in d. **f** The basic protein expression of BATF2 in normal gastric epithelial cells (GES-1) and GC cell lines (HGC-27, MGC-803, AGS, MKN-45 and SNU-216) was detected by western blotting and quantified (**P < 0.01; ***P < 0.001). **g** The expression of BATF2 in 352 paraffin-embedded specimens from the internal cohort was determined by TMA-based IHC staining. Scale bars = 200 μm. **h**-**i** Kaplan-Meier analyses of the correlations between BATF2 expression and overall survival or disease-free survival in the internal cohort (*P* < 0.05). **j** The BATF2 IHC scores in the internal cohort are shown as box plots. A negative correlation was detected between the BATF2 IHC scores in GC and the frequency of peritoneal recurrence (***P < 0.001; P-rec: peritoneal recurrence). **k** BATF2 IHC scores in paired normal and gastric tumor tissues according to disease stage and the presence or absence of peritoneal recurrence (***P < 0.001; P-rec: peritoneal recurrence). **l** The cumulative incidence of peritoneal recurrence in GC patients with different BATF2 expression levels from the internal cohort (*P* < 0.05)

To identify dysregulated genes in GC, we performed transcriptome sequencing in 8 pairs of GC and normal gastric tissues and analyzed the available clinical data (TCGA-STAD and ACRG datasets) (Fig. 1a). BATF2 was identified as a downregulated gene and selected for subsequent experiments (Fig. 1b). We found that BATF2 expression was lower in GC tissues than in normal gastric tissues at both mRNA (Fig. 1c) and protein levels (Fig. 1d-e). Moreover, in GC cell lines, BATF2 expression was also decreased compared to that in normal gastric cells (Fig. 1f and Additional file 3: Figure S1A).

We further evaluated the prognostic role of BATF2 in GC using IHC (Fig. 1g). A sharp contrast in BATF2 staining was observed between the tumor and nontumor tissues (Additional file 3: Figure S1B-C). BATF2 expression was scored as low in 257 (73.0%) and high in 95 (27.0%) samples. The decreased BATF2 IHC expression was associated with sex, tumor size, histological grade and TNM stage (Table 1). The overall and disease-free survival for patients with low BATF2 expression were significantly poorer than those for patients with high BATF2 expression (Fig. 1h-i). Multivariate Cox regression analysis demonstrated that BATF2 expression was an independent prognostic factor for overall survival of patients with GC (Additional file 4: Table S2). Next, we analyzed recurrence patterns based on BATF2 expression in GC patients who underwent curative gastrectomy. Of 352 patients, 127(36.1%) experienced postoperative recurrence. Analysis of the recurrence patterns showed that low BATF2 expression was significantly associated with peritoneal recurrence (*P* = 0.012) (Additional file 5: Table S3), but not with the other recurrence patterns. BATF2 expression was scored as much higher in tissues without peritoneal recurrence than in those with peritoneal recurrence (Fig. 1j). The level of BATF2 was lower in stage III GC patients who suffered peritoneal recurrence than in those who did not (Fig. 1k). The cumulative incidence of peritoneal recurrence was higher in the BATF2-low group than in the BATF2-high group (Fig. 1l). Based on the multivariate analysis, BATF2 expression was identified as an independent risk factor for peritoneal recurrence (Additional file 6: Table S4). To confirm whether BATF2 had the same excellent prognostic value in different populations, we further evaluated it to the external validation cohort and obtained similar results (Additional file 3: Figure S1D-I, Table 1, Additional file 4: Table S2 and Additional file 6: Table S4).

**Table 1:** Correlation between BATF2 expression and clinicopathological characteristics of GC patients

Variables	Internal cohort				External validation cohort			
	BATF2 low (*n* = 257)	BATF2 high (*n* = 95)	χ^2^	*P*	BATF2 low (*n* = 166)	BATF2 high (*n* = 66)	χ^2^	*P*
Age (years)			0.321	0.571			6.426	0.011*
< 65	152	53			99	51		
≥ 65	105	42			67	15		
Sex			4.504	0.034*			0.516	0.472
Female	58	32			48	16		
Male	199	63			118	50		
BMI			0.333	0.564				
≤ 25	215	77						
> 25	42	18						
Tumor size (mm)			8.150	0.004*			0.003	0.953
< 50	121	61			110	44		
≥ 50	136	34			56	22		
Tumor location			5.238	0.155			6.130	0.105
Upper	83	22			40	21		
Middle	46	14			66	31		
Low	103	44			57	12		
Overlap	25	15			3	2		
Histological grade			5.180	0.023*			0.815	0.367
Well/Moderately	83	43			55	26		
Poor	151	44			111	40		
TNM stage			5.740	0.017*			7.758	0.005*
I&II	86	45			72	42		
III&IV	171	50			94	24		

*P < 0.05 was considered significant

### BATF2 inhibits GC growth in vitro and in vivo

**Fig. 2:**
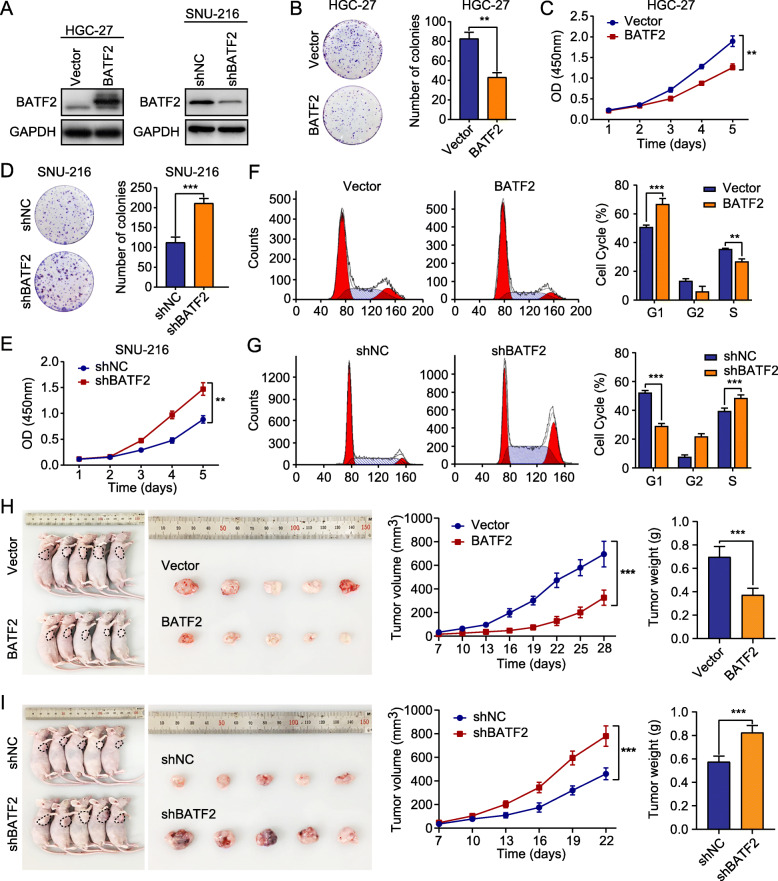
BATF2 inhibits GC growth in vitro and in vivo. **a** HGC-27 cells with stable BATF2 overexpression or SNU-216 cells with BATF2 knockdown were created. The changes in BATF2 expression were confirmed using western blotting. **b-e** The proliferative ability of stably transfected HGC-27 or SNU-216 cells was investigated via colony formation and CCK-8 assays (**P < 0.01). **f-g** Flow cytometry analysis of stably transfected HGC-27 or SNU-216 cells was performed. Representative images and quantification of the results are presented (**P < 0.01; ***P < 0.001). **h** Overexpression of BATF2 inhibits GC growth in a subcutaneous xenograft model. The size of the tumors was measured at the indicated time points (***P < 0.001). Tumors were extracted and weighed after mice were sacrificed (***P < 0.001). **i** BATF2 knockdown promotes GC growth in a subcutaneous xenograft model. The size of the tumors was measured at the indicated time points (***P < 0.001). Tumors were extracted and weighed after mice were sacrificed (***P < 0.001)

**Fig. 3:**
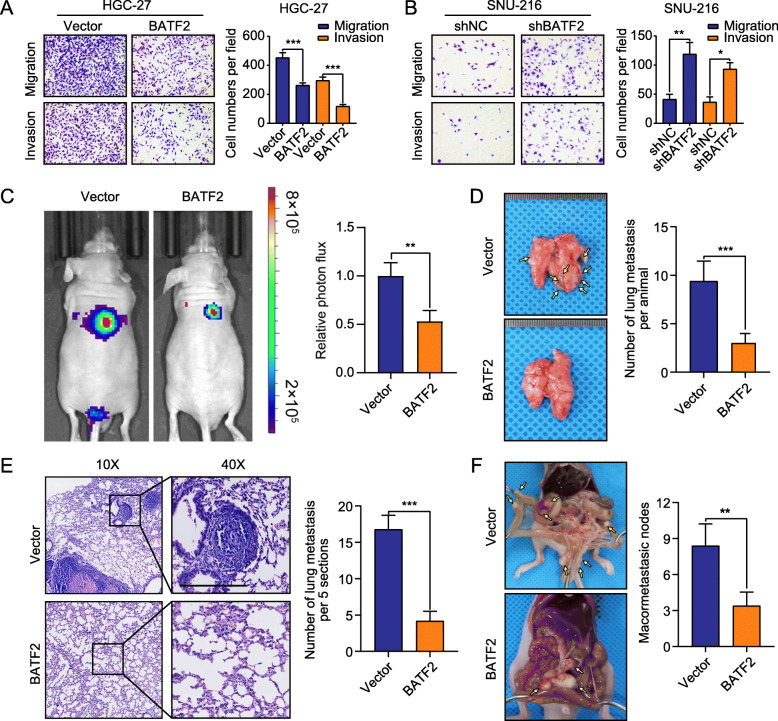
BATF2 suppresses GC metastasis in vitro and in vivo. **a-b** Transwell assays with stably transfected HGC-27 and SNU-216 cells were performed. Representative images and quantification of the results are presented (*P < 0.05; **P < 0.01; ***P < 0.001). **c** Representative bioluminescence images of mice at 4 weeks after tail vein injection of BATF2-overexpressing HGC-27 cells or control cells and quantification of the images are presented. **d-e** Representative images of lung metastasis and H&E (hematoxylin and eosin) staining are shown. Metastatic nodules were counted with or without a microscope and recorded. Overexpression of BATF2 in HGC-27 cells significantly reduced the number of metastatic lesions in the lungs (***P < 0.001). Scale bars = 200 μm. **f** Stably transfected HGC-27 cells were injected intraperitoneally, and the number of metastases in the colonic wall was recorded 4 weeks later. Peritoneal metastases were examined and recorded (**P < 0.01)

To examine the function of BATF2 in GC, we established GC cell lines (HGC-27, MGC-803, and SNU-216) with stable overexpression or knockdown of BATF2 (Fig. 2a and Additional file 7: Figure S2A). Upregulation of BATF2 significantly suppressed GC cell proliferation, while reduced BATF2 expression resulted in increased cell proliferation (Fig. 2b-e and Additional file 7: Figure S2B-C). Flow cytometric assessment of cell cycle distribution revealed that ectopic BATF2 expression increased the proportion of cells in G1 phase (Fig. 3f), whereas BATF2 knockdown resulted in the opposite trend (Fig. 3g). To evaluate the effect of BATF2 in vivo, xenograft animal models were used. Consistent with the in vitro results, xenografts with high BATF2 expression grew more slowly than did the control xenografts (Fig. 2h), whereas xenografts with downregulated BATF2 expression grew much faster than the control xenografts (Fig. 2i). IHC staining of Ki-67 was scored as lower in the BATF2-high group than in the BATF2-low group (Additional file 7: Figure S2D). These data suggested that BATF2 may inhibit GC growth by regulating the cell cycle.

### BATF2 modulates GC metastasis in vitro and in vivo

**Fig. 4:**
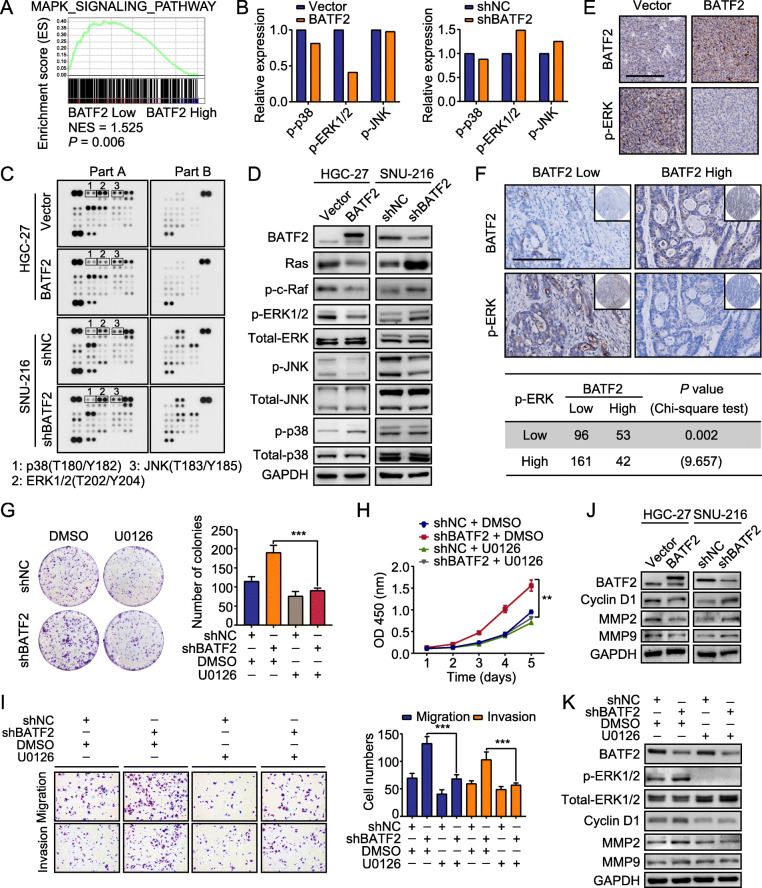
Identification of extracellular signal-regulated kinase (ERK) as a regulatory target of BATF2. **a** Gene set enrichment analysis (GSEA) identified a significant association between BATF2 and the MAPK signaling pathway. **b-c** Human phospho-kinase microarray assay analysis of the conditioned medium from stably transfected HGC-27 and SNU-216 cells. A summary of the relative signal intensities of the indicated proteins is shown. **d** The expression of critical members of the MAPK signaling pathway was examined by western blotting in stably transfected HGC-27 and SNU-216 cells. **e** Representative images of BATF2 and p-ERK IHC staining in xenograft samples. Scale bars = 200 μm. **f** IHC staining of BATF2 and p-ERK in TMAs were presented, and their correlation was calculated. Scale bars = 200 μm. **g-i** The effect of BATF2 downregulation on SNU-216 cell proliferation, migration and invasion was rescued by treatment with U0126 (10 μM) (**P < 0.01; ***P < 0.001). **j** The expression of downstream effectors of ERK signaling was examined by western blotting in stably transfected HGC-27 and SNU-216 cells. **k** The protein levels of cyclin D1, MMP2, and MMP9 in stably transfected SNU-216 cells treated with U0126 (10 μM) or DMSO (control) were determined by western blotting

To determine the role of BATF2 in GC metastasis, we first performed cell migration and invasion assays in vitro. The results showed that ectopic expression of BATF2 significantly suppressed the migration and invasion of HGC-27 and MGC-803 cells (Fig. 3a and Additional file 8: Figure S3A-C). Conversely, BATF2 knockdown in HGC-27 and SNU-216 cells elicited the opposite effects (Fig. 3b and Additional file 8: Figure S3D-F). We further assessed the effect of BATF2 on GC metastasis in vivo. HGC-27 cells overexpressing BATF2 or corresponding control cells were injected into nude mice via tail vein. When investigating the lungs at 4 weeks after injection, mice injected with BATF2-overexpressing cells exhibited significantly reduced GC lung metastasis, as shown by bioluminescence imaging (Fig. 3c). The number of metastatic colonies in the control group indicated enhanced invasion compared with that in the BATF2-overexpression group (Fig. 4d). Histological analysis by H&E staining of the dissected lungs confirmed that the control group had more metastatic nodules than did the BATF2-overexpression group (Fig. 4e). Furthermore, enhanced BATF2 expression significantly inhibited peritoneal metastasis after intraperitoneal injection of GC cells (Fig. 4f). Collectively, these results indicate the critical role of BATF2 in suppressing GC metastasis.

### BATF2 suppresses GC via ERK signaling

We next investigated the molecular mechanism by which BATF2 inhibits GC progression. Gene set enrichment analysis (GSEA) showed that BATF2 was correlated with the MAPK signaling pathway (Fig. 4a). Human phospho-kinase microarray assays were performed to examine the signaling impact of BATF2. As shown in Fig. 4b-c, BATF2 knockdown significantly enhanced ERK phosphorylation (Thr202/Tyr204), whereas overexpression of BATF2 decreased ERK phosphorylation. Western blot assay also confirmed changes in p-ERK protein levels when BATF2 was upregulated or downregulated (Fig. 4d and Additional file 9: Figure S4A). IHC staining of p-ERK in xenograft samples was significantly weaker in the BATF2-overexpression group than in the respective control group (Fig. 4e). Furthermore, TMA-based IHC validated that 62.6% of patients in the low BATF2 expression group exhibited enhanced p-ERK levels (Fig. 4f).

The effect of p-ERK on BATF2 function in GC cells was then explored using U0126 (a MEK/ERK-specific inhibitor). Inhibition of p-ERK in BATF2-knockdown SNU-216 cells abrogated the increased colony formation (Fig. 4g) and accelerated proliferation (Fig. 4h) and migration and invasion (Fig. 4i and Additional file 9: Figure S4B) of cells. Furthermore, we analyzed the ERK-mediated transcriptional targets MMP2, MMP9, and cyclin D1 and confirmed that the expression of MMP2, MMP9 and cyclin D1 was decreased in BATF2-overexpressing cells and increased BATF2-knockdown cells (Fig. 4j). In stable SNU-216 cells, U0126 efficiently reduced the levels of p-ERK and its downstream targets, even when BATF2 was knocked down (Fig. 4k).

To evaluate the clinical prognostic value of the combined BATF2 and p-ERK expression, we assessed the overall survival of GC patients categorized according to BATF2 and p-ERK expression in the internal cohort. We discovered that the prognostic value of BATF2 was dependent on p-ERK expression (Additional file 9: Figure S4C). The impact of BATF2 and p-ERK on the prognosis of patients in the external validation cohort was similar to that in the internal cohort (Additional file 9: Figure S4D). These data indicate that BATF2-mediated changes in ERK phosphorylation may be the mechanism underlying the tumor-suppressive function of BATF2 in GC.

### BATF2 controls ERK phosphorylation in the presence of p53

**Fig. 5:**
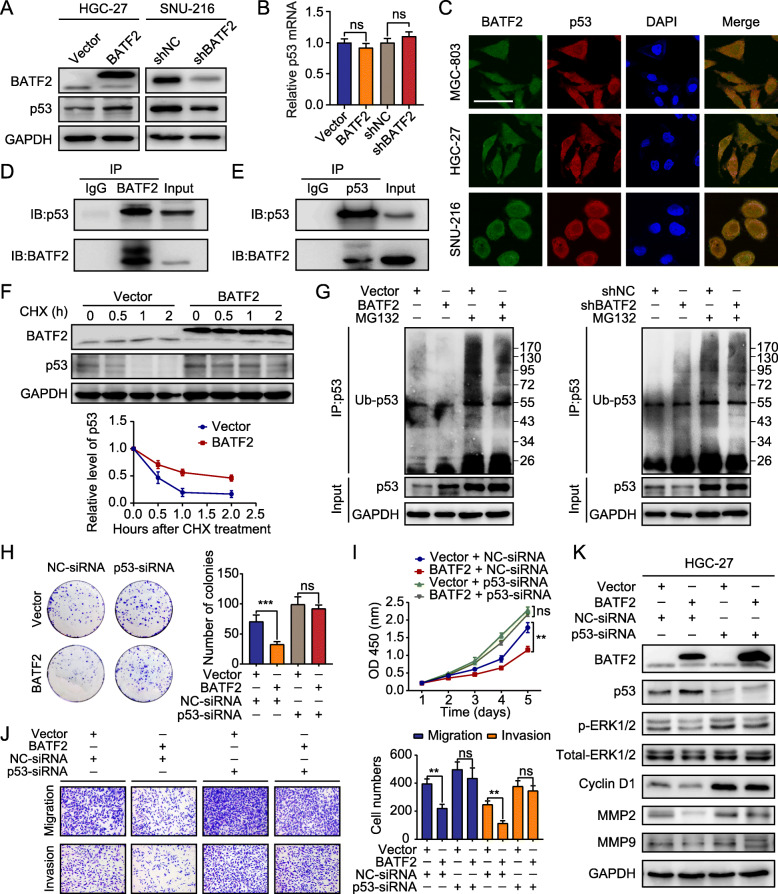
p53 is critical for BATF2-mediated inhibition of ERK signaling. **a** p53 expression in stably transfected HGC-27 and SNU-216 cells was determined by western blotting. **b** p53 expression in stably transfected HGC-27 and SNU-216 cells was determined by qRT-PCR (ns: no significant difference). **c** Immunofluorescence (IF) staining indicating the colocalization of BATF2 (green) and p53 (red) together with DAPI (blue) in GC cells. Scale bars = 100 μm. **d-e** A co-immunoprecipitation (co-IP) assay was performed to analyze the interaction between BATF2 and p53 in SNU-216 cells. **f** The half-life of p53 protein in HGC-27 cells was analyzed following treatment with cycloheximide (CHX, 25 μg/ml) at the indicated time points. **g** Stably transfected HGC-27 and SNU-216 cells were treated with or without 10 mmol/l MG132 for 6 h, p53 was immunoprecipitated with an anti-p53 antibody, and the poly-ubiquitination of p53 was examined by western blotting using an anti-ubiquitin antibody. IP: immunoprecipitation; Ub-p53: poly-ubiquitinated of p53. **h-j** The effect of BATF2 overexpression on HGC-27 cell proliferation, migration and invasion was rescued by transfection with p53 siRNA (**P < 0.01; ns: no significant difference). **k** The protein levels of p53, p-ERK, MMP2, MMP9 and cyclinD1 in stably transfected HGC-27 cells treated with p53-siRNA or NC-siRNA were determined by western blotting

We analyzed the potential proteins that interact with BATF2 in the Search Tool for the Retrieval of Interacting Genes (STRING) database. We noticed that p53 protein, might interact with BATF2 (Additional file 10: Figure S5A). Interestingly, BATF2 overexpression resulted in increased p53 protein expression, whereas silencing BATF2 decreased p53 expression (Fig. 5a). IHC staining of xenograft samples also showed that p53 protein was positively correlated with BATF2 expression (Additional file 10: Figure S5B). However, qRT-PCR assays showed no significant correlation between BATF2 and p53 expression at the transcriptional level (Fig. 5b). Using immunofluorescence staining, we also found that BATF2 colocalized with p53 in GC cells (Fig. 5c). The co-IP assays revealed that BATF2 and p53 protein interacted with each other (Fig. 5d-e). Therefore, we hypothesized that BATF2 might regulate the protein level of p53 via posttranslational modification.

Subsequently, we investigated whether BATF2 affected p53 expression by regulating its protein stability. BATF2 overexpression dramatically increased the half-life of p53 protein after the addition of the translation inhibitor cycloheximide (CHX) (Fig. 5f). MG132, a proteasome inhibitor, rescued the change in p53 protein levels induced by BATF2 overexpression (Fig. 5g).

We also demonstrated that silencing p53 in GC cells restored their proliferative and metastatic capacities (Fig. 5h-j and Additional file 10: Figure S5C). Silencing p53 expression significantly abolished the inhibitory effect of BATF2 on the levels of p-ERK, MMP2, MMP9 and cyclin D1 (Fig. 5k). Taken together, these results indicate that the loss of BATF2 promotes ubiquitination-mediated degradation of p53 and consequently activates ERK signaling.

### METTL3-mediated m^6^A modification of BATF2 mRNA in GC

**Fig. 6:**
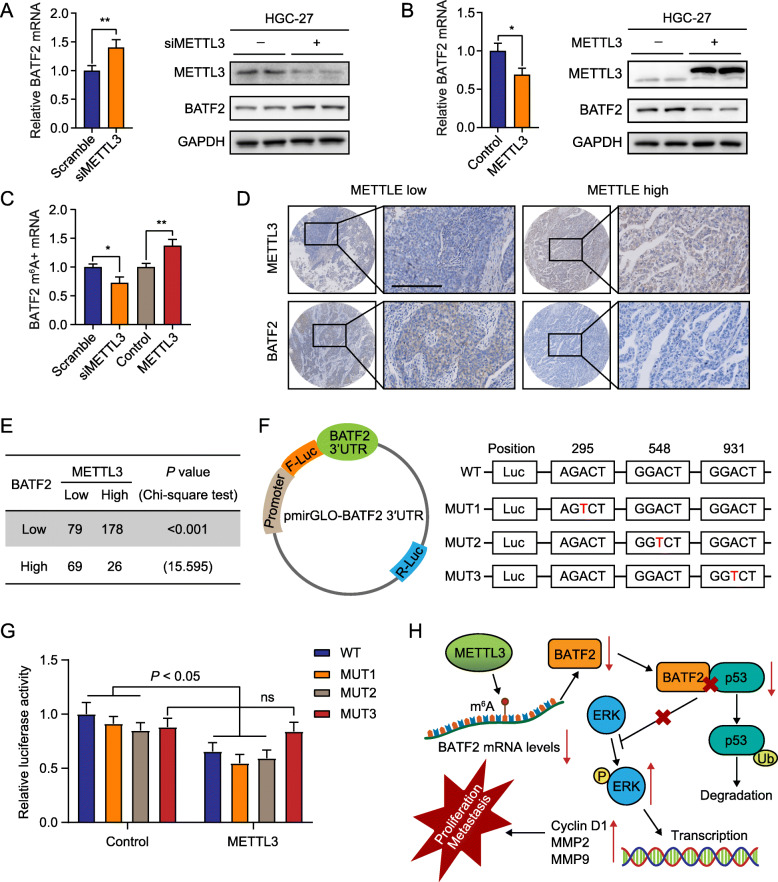
METTL3-mediated m^6^A modification represses BATF2 expression in GC. **a-b** qRT-PCR and western blot assays showed the mRNA and protein expression, respectively, of BATF2 in GC cells with knockdown or overexpression of METTL3 (*P < 0.05; **P < 0.01). **c** m^6^A immunoprecipitation and qRT-PCR assays showed the relative percentage of BATF2 mRNA with methylation (*P < 0.05; **P < 0.01). **d-e** IHC staining of BATF2 and METTL3 in TMAs were presented, and their correlation was calculated. Scale bars = 200 μm. **f** Wild-type BATF2 3′UTR and BATF2 3′UTR with a mutation at the m^6^A consensus sequence were cloned into a luciferase reporter. Mutations of the m^6^A modification region were generated by replacing adenosine with thymine. **g** Relative luciferase activity of the wild-type and 3 mutant BATF2 3′UTR reporter vectors catalyzed by METTL3 (ns: no significant difference). **h** Proposed mechanism scheme of BATF2 in GC. The m^6^A modification of BATF2 mRNA by METTL3 represses its expression; BATF2 promotes the expression of p53 by suppressing its ubiquitination and degradation, which further decreased ERK signaling

The mechanisms leading to aberrant BATF2 expression remain unclear. Considering the low mRNA level of BATF2 in GC, we wondered whether N6-methyladenosine (m^6^A) modification regulates the mRNA stability of BATF2. Silencing the expression of METTL3, an important m^6^A methyltransferase, increased BATF2 expression, and vice versa (Fig. 6a-b). We found that the m^6^A modification of BATF2 mRNA was positively correlated with METTL3 expression in GC cells (Fig. 6c). Furthermore, TMA-based IHC showed that METTL3 expression was negatively correlated with BATF2 in GC to some extent (Fig. 6d-e).

The online prediction tool SRAMP revealed that numerous m^6^A sites with high confidence are distributed in the 3′ untranslated region (3′UTR) of BATF2. We then constructed three mutant BATF2 3′UTR plasmids for the luciferase reporter assay to determine the specific modification sites (Fig. 6f). As shown in Fig. 6g, METTL3 modulated BATF2 expression mainly through the MUT3 site but neither the MUT1 nor MUT2 sites. These results demonstrated that m^6^A maintained the mRNA stability of BATF2 by METTL3 in GC.

In addition, 96 GC patients were tested for *H. pylori* infection (30 cases in the *H. pylori*-positive group, 66 cases in the *H. pylori*-negative group), and we found that BATF2 and METTL3 IHC expression in the two groups was not significantly different (Additional file 11: Figure S6A). At the same time, Spearman correlation analysis revealed that there was no correlation between *H. pylori* infection and either BATF2 or METTL3 expression in GC tissues (*r* = − 0.031 and *r* = 0.031, respectively, both *P* > 0.05). We obtained similar results in the ACRG dataset (Additional file 11: Figure S6B, r = 0.045, *r* = − 0.064, respectively, both *P* > 0.05).

## Discussion

Recent discoveries have indicated the critical roles of BATF2 in the pathogenesis of cancers. However, the biological function of BATF2 and its regulatory mechanisms in GC are still unclear. Our study demonstrated that BATF2 is expressed at low levels in human GC tissues compared with that in matched normal tissues, and low BATF2 expression was closely correlated with poor prognosis of GC patients based on 2 cohorts. Moreover, we first revealed that BATF2 binds to p53 and enhances its protein stability, which can inhibit ERK signaling to suppress GC growth and metastasis. Additionally, the m^6^A modification of BATF2 mRNA by METTL3 repressed its expression in GC (Fig. 6h).

Accumulating evidence has shown that BATF2 plays an important role in cell cycle regulation, EMT, inflammatory responses, and resistance to therapies [7, 16, 17]. In accordance with previous studies, we found that BATF2 acts as a multifunctional protein that inhibits GC growth and metastasis both in vitro and in vivo. Notably, BATF2 arrested the G0/G1 to S phase transition, while knockdown of BATF2 induced cell cycle progression; this could partially explain why BATF2 is associated with GC cell proliferation. Thus, we confirmed that BATF2 acts as a tumor suppressor in GC.

Multiple different signaling pathways, including the Wnt/β-catenin, PI3K/Akt, ERK, and NF-κB, have been reported to regulate cancer progression [18, 19]. ERK is one of the most vital pathways that contributie to GC growth and metastasis [20, 21]. One potential mechanism by which ERK signaling could increase the migration, invasion, and proliferation of GC cells is by elevating the expression of MMPs and cyclin D1 [22]. MMP2 and MMP9 are closely associated with cancer metastasis due to their strong proteolytic activity of the ECM (extracellular matrix) [23]. Cyclin D1, a vital regulatory target of G1-S phase transition, is frequently constitutively expressed and is associated with pathogenesis in tumors [24]. In the present study, we demonstrated that the role of BATF2 in GC is mediated through the inactivation of ERK signaling, which results in the downregulation of the expression of MMP2, MMP9, and cyclin D1.

It has been investigated that the foundation of the carboxyterminal domain of BATF2 (amino acids 80 to 274) involved in the interaction between BATF2 and ceruloplasmin [9], which suggests that the carboxyterminal domain of BATF2 interacts with other proteins. Our study first indicated that BATF2 binds to p53 and attenuates its ubiquitination-mediated degradation. Furthermore, it has been reported that p53 can regulate ERK signaling in various cancers [25–27]. Here, we proved that BATF2 interacted with p53, which consequently inhibited the activation of ERK signaling. Our future studies will aim to identify the exact binding site of BATF2 that interacts with p53 and the molecular basis by which p53 regulates ERK phosphorylation in GC.

Currently, over 100 types of chemical modifications have been found in human RNA, among which the m^6^A modification is the most prevalent in mRNA and non-coding RNA [28, 29]. The m^6^A modification is regulated by three types of proteins: writers (METTL3, METTL14 and WTAP), erasers (FTO, ALKBH5) and readers (YTHDFs) [30]. Among these enzymes, METTL3 is involved in cancer progression by regulating cancer-related gene expression. Here, we found that METTL3 mediated the m^6^A modification of BATF2 mRNA and suppressed its expression. However, whether METTL3 induces mRNA alternative splicing or affects the mRNA stability of BATF2 remains to be further studied.

## Conclusion

In summary, our current findings reveal a tumor suppressor role of BATF2 in GC development. Mechanistically, the METTL3/BATF2/p53/ERK axis inhibits GC growth and metastasis by regulating cell cycle progression and ECM degradation. Moreover, significant downregulation of BATF2 expression is correlated with poor prognosis of patients with GC. Thus, BATF2 might be a potential predictor and therapeutic target for GC.

## Supplementary information

**Additional file 1.** Supplementary Materials and Methods.

**Additional file 2: Table S1.** Primers used for qRT-PCR.

**Additional file 3: Figure S1.** The expression and prognostic value of BATF2 in GC. **a** The mRNA levels of BATF2 in human normal gastric epithelial cells (GES-1) and human GC cell lines (HGC-27, MGC-803, AGS, MKN-45 and SNU-216) were detected by real-time PCR and quantified (***P < 0.001). **b** Representative images of BATF2 IHC staining in gastric tumor and adjacent normal tissues. **c** For the internal cohort, BATF2 IHC scores in gastric tumor and adjacent normal tissues are shown as box plots (***P < 0.001). **d** The expression of BATF2 in 232 paraffin-embedded specimens from the external validation cohort was determined by TMA-based IHC staining. Scale bars = 200 μm. **e-f** Kaplan-Meier analyses of the correlations between BATF2 expression and overall survival or disease-free survival in the external validation cohort. **g** BATF2 IHC scores in the external validation cohort are shown as box plots. A negative correlation was detected between the BATF2 IHC scores in GC and the frequency of peritoneal recurrence (**P < 0.01; P-rec: peritoneal recurrence). **h** BATF2 IHC scores in the external validation cohort according to disease stage and the absence or presence of peritoneal recurrence (**P < 0.01; P-rec: peritoneal recurrence). **i** The cumulative incidence of peritoneal recurrence in GC patients with different BATF2 expression levels from the external validation cohort.

**Additional file 4: Table S2.** Univariate and multivariate analyses of overall survival for GC patients

**Additional file 5: Table S3.** Analysis of the recurrence sites of GC after curative gastrectomy

**Additional file 6: Table S4.** Univariate and multivariate analyses of peritoneal recurrence after gastrectomy

**Additional file 7: Figure S2.** The effect of BATF2 expression on GC cell proliferation. **a** MGC-803 and HGC-27 cells with stable BATF2 overexpression or knockdown were created. The changes in BATF2 expression were confirmed using western blotting. **b** Colony formation and CCK-8 assays with stably transfected MGC-803 cells were performed. Representative images and quantification of the results are presented (**P < 0.01; ***P < 0.001). **c** Colony formation and CCK-8 assays with stably transfected HGC-27 cells were performed. Representative images and quantification of the results are presented (*P < 0.05; **P < 0.01). **d** Representative images of Ki-67 IHC staining in xenografted tumors. Scale bars = 200 μm. The Ki-67 IHC scores are shown in the indicated tissues (**P < 0.01).

**Additional file 8: Figure S3.** The effect of BATF2 expression on GC cell invasion and migration. **a-b** Transwell and wound healing assays with stably transfected MGC-803 cells were performed. Representative images and quantification of the results are presented (**P < 0.01; ***P < 0.001). **c-e** Transwell and wound healing assays with stably transfected HGC-27 cells were performed. Representative images and quantification of the results are presented (**P < 0.01; ***P < 0.001). **f** Wound healing assays with stably transfected SNU-216 cells were performed. Representative images and quantification of the results are presented (***P < 0.001).

**Additional file 9: Figure S4.** The clinical value of BATF2 depends on ERK activity. **a** The expression of critical members of the MAPK pathway in stably transfected MGC-803 cells was examined by western blotting. **b** Wound healing assays showed that the effect of BATF2 downregulation on SNU-216 cell migration was rescued by U0126 treatment. Representative images are presented (**P < 0.01). **c** Kaplan-Meier analyses of the correlations between BATF2 and p-ERK expression in the internal cohort. **d** Kaplan-Meier analyses of the correlations between BATF2 and p-ERK expression in the external validation cohort.

**Additional file 10: Figure S5.** The relationship between BATF2 and p53. **a** The online tool STRING was used to predict potential protein-protein interactions. **b** Representative images of p53 IHC staining in xenograft samples. **c** Wound healing assays showed that the effect of BATF2 overexpression on HGC-27 cell migration was rescued by p53 siRNA transfection. Representative images are presented (*P < 0.05; ns: no significant difference).

**Additional file 11: Figure S6.** Relative expression of BATF2 and METTL3 in GC shown by *H. pylori* status. **a** BATF2 and METTL3 IHC scores from the internal cohort. **b** Relative expression of BATF2 and METTL3 from the ACRG dataset (GSE62254). ns: no significant difference.

## Data Availability

The datasets used and/or analyzed during the current study are available from the corresponding author on reasonable request.

## Abbreviations

BATF2: Basic leucine zipper ATF-like transcription factor 2
GC: Gastric cancer
ACRG: Asian Cancer Research Group
TCGA-STAD: The Cancer Genome Atlas Stomach Adenocarcinoma
GEO: Gene Expression Omnibus
qRT-PCR: Quantitative reverse transcription PCR
IHC: Immunohistochemistry
TMA: Tissue microarray
H&E: Hematoxylin and eosin
AJCC: American Joint Committee on Cancer
HR: Hazard ratios
GSEA: Gene set enrichment analysis
ERK: Extracellular signal-regulated kinase
ECM: Extracellular matrix
STRING: Search Tool for the Retrieval of Interacting Genes
CHX: Cycloheximide
m^6^A: N6-methyladenosine
METTL3: Methyltransferase-like 3
WT: Wild-type
MUT: Mutant-type
3′UTR: 3′ untranslated region
