# Supplementary material for: m6A modification-mediated BATF2 acts as a tumor suppressor in gastric cancer through inhibition of ERK signaling
Source: Mol Cancer. 2020 Jul 10;19:114. doi: 10.1186/s12943-020-01223-4 (PMC7350710; doi:10.1186/s12943-020-01223-4)
Supplement: Supplementary file 1 — Additional file 1. Supplementary Materials and Methods. [file 12943_2020_1223_MOESM1_ESM.docx]

**Supplementary** **Materials and Methods**

**Immunohistochemistry (IHC)**

Paraffin blocks containing formalin-fixed tumor specimens were serially sectioned at a thickness of 4 μm and mounted on silane-coated slides for IHC analysis. The sections were deparaffinized with dimethylbenzene and rehydrated with a graded series of ethanol (100, 95, 85 and 75% ethanol). Antigen retrieval was performed with 0.01 mol/l sodium citrate buffer (pH 6.0) in an autoclave at 121°C for 2 minutes, and endogenous peroxidase was blocked by incubating the sections with 3% hydrogen peroxide for 10 minutes at room temperature. The sections were then washed in phosphate-buffered saline (PBS), blocked with 10% goat serum (ZhongShan Biotechnology, China) for 30 minutes and incubated with antibodies targeting BATF2 (ab204510; Abcam, Cambridge, MA) in a humidified chamber at 4°C overnight. Following three washes in PBS, the sections were incubated with HRP-conjugated secondary antibody (ZhongShan Biotechnology, China) for 30 minutes at room temperature. Next, the signal was developed with diaminobenzidine (DAB) solution (ZhongShan Biotechnology, China), and all the sections were counterstained with 20% hematoxylin. Finally, the sections were dehydrated and mounted with cover slips. For negative controls, the primary antibody diluent was used instead of primary antibody. The staining intensity was scored as 0 to 3. The heterogeneity of staining was scored as 0 to 3, depending on the percentage of tumor cells that were positively stained. To obtain an IHC score that considers the signal intensity and staining frequency, we generated a composite expression score (CES) ranging from 0 to 9 by multiplying the intensity and heterogeneity scores. A CES of 0, 1, 2 and 3 was defined as low expression, and a CES of 4, 6 and 9 was defined as high expression.

**Cell culture**

Five GC cell lines (HGC-27, MGC-803, MKN-45, AGS, and SNU-216) and one normal gastric epithelial cell line (GES-1) were used in this study. HGC-27, MKN-45 and AGS cells were obtained from CellCook Biotech Co., Ltd. (Guangzhou, China). MGC-803 and GES-1 cells were obtained from the Cell Line Bank, Chinese Academy of Sciences. The SNU-216 cell line was a gift from Dr. Jun Lin (College of Biological Science and Engineering, Fuzhou University, Fuzhou, China). All the cell lines were confirmed to be free of mycoplasma contamination by PCR and culture, and the species origin was confirmed by PCR. The identities of the cell lines were authenticated with short tandem repeat (STR) profiling. GES-1, HGC-27, MGC-803, MKN-45 and SNU-216 cells were cultured in 1640 medium (Gibco, Grand Island, NY), and whereas AGS cells were cultured in DMEM/F12 (Gibco, Grand Island, NY); all media were supplemented with 10% fetal bovine serum (FBS) (Gibco, Grand Island, NY), and all cells were incubated at 37°C in a humidified atmosphere containing 5% CO_2_.

**Quantitative real-time PCR**

Total RNA was extracted from cell lines and tissue samples using TRIzol Reagent (Invitrogen, Carlsbad, CA, USA). First-strand cDNA was synthesized with PrimeScript RT Master Mix (Takara Biotechnology, Ltd., Dalian, China), according to the manufacturer’s protocol. After reverse transcription of total RNA, qPCR was conducted to examine the expression levels of BATF2 using SYBR Green PCR Master mix (Takara Biotechnology, Ltd.) on a Bio-Rad Real-Time PCR instrument (Bio-Rad Laboratories, Inc., Hercules, CA, USA). GAPDH was used as an internal reference gene to normalize the mRNA levels between different samples for an exact comparison of transcription levels. The primers used in our study are listed in Additional file 2: Table S1. Data were analyzed using the ΔΔCt method with GAPDH as the constitutive marker [1].

**Western blotting**

Cells were plated into 60-mm dishes and cultured to 80% confluence. The cells were then scraped and lysed in RIPA buffer and the lysates were centrifuged at 10,000 × g (4°C for 10 minutes). Protein concentrations were determined using a BCA Protein Assay Kit (Thermo) according to the manufacturer’s instructions. A total of 40 µg protein from each sample was denatured, loaded into a well in a polyacrylamide gel, separated by SDS-PAGE, and transferred to a polyvinylidene difluoride membrane (Millipore, Billerica, MA). The membranes were blocked with 5% nonfat milk at room temperature for one hour and incubated overnight with primary antibodies in PBST (1:1000). After the membranes were washed with PBST, they were incubated for 1 hour at room temperature with the corresponding horseradish peroxidase-conjugated secondary antibody at the appropriate dilution and then washed three times with PBST. The protein bands on the membranes were detected using enhanced chemiluminescence (Amersham Corporation, Arlington Heights, IL, USA). Antibodies against BATF2 (ab157466), cyclin D1 (ab134175), p53 (ab1101), and GAPDH (ab181602) were obtained from Abcam (Cambridge, MA), and antibodies against ERK (#4695), p-ERK (#4370), JNK (#9252), p-JNK (#4668), p38 (#9212) and p-p38 (#4511) were purchased from Cell Signaling Technology (Danvers, MA). Antibodies targeting METTL3 (DF12020), MMP2 (AF0577) and MMP9 (AF5228) were purchased from Affinity Biosciences (Cincinnati, OH). Horseradish peroxidase-conjugated goat anti-rabbit IgG (A4914) and goat anti-mouse IgG(A0168) were purchased from Sigma. We corrected the loading error by staining for and quantifying loading controls and made comparisons of the expression levels of target proteins between tumor and normal tissues. Protein expression in tumors was defined as high when it was higher than that in normal tissue but was defined as low when it was lower than that in normal tissue.

**Cell proliferation**

For the CCK-8 assay, 1 × 10^3^ cells in the logarithmic growth phase were seeded in 96-well plates. In brief, 10 ul of CCK-8 in 1640 medium (100 μl) was added to each well, and the cells were incubated for 2 hours at 37°C. Then, the optical density values were measured at 450 nm on a microplate reader (Bio-Rad, Hercules, CA, USA). The cell growth rate was examined at 0, 1, 2, 3 and 4 days after seeding, and statistical results were obtained from three independent experiments. For the colony formation assay, 1×10³ cells were seeded in 6-well plates and cultured for 7-14 days. Media were changed every other day, and colonies were stained with 0.5% crystal violet for 20 minutes. Only colonies containing more than 50 cells were counted. Statistical results were obtained from three independent experiments.

**Flow cytometry**

A total of 5 × 10^5^ cells was seeded in 6 cm plates and incubated for 24 hours. Next, cells were harvested and fixed with 70% ethanol at 4°C overnight. After the cells were washed, they were stained with propidium iodide (PI) and then filtered through a 70 μm cell strainer immediately prior to flow cytometry, which was carried out on a FACSVerse flow cytometer (BD Biosciences, San Jose, CA).

**Cell migration and invasion assays**

Transwell assays were performed using transwell chambers (polycarbonate filters with 8 μm pores, BD Bioscience). A total of 1×10^5^ cells in 200 μl of serum-free medium were seeded in the upper chamber, and 500 μl of culture medium containing 10% FBS was added to the lower chamber. After 12-24 hours of incubation, cells that migrated or invaded to the bottom chamber were stained with 0.5% crystal violet. Cells were counted in three randomly selected fields (magnification, × 200) per well. For the wound healing assay, 5 × 10^5^ cells were seeded in 6-well plates and grown to 100% confluence. Wounds were created by scraping the confluent cell monolayers with a 200 μl pipette tip. After the plates were extensively rinsed to remove cellular debris, serum-free medium was added. The wound closure rate was recorded at 0, 24 or 48 hours under a 20× objective lens, and the wound healing rate was quantified as the width of wound at the indicated time point versus that of the wound at time 0. Statistical results were obtained from three independent experiments.

**Co-immunoprecipitation (co-IP) assay**

Cells were washed with PBS and lysed in Tris-buffered saline (pH 7.4). Lysates were incubated on ice for 30 minutes before cellular debris and nuclei were removed via centrifugation at 10,000 × g for 5 minutes. Cell lysates were incubated with BATF2 antibody and p53 antibody overnight at 4°C. Protein A-Sepharose (Amersham Biosciences, Piscataway, NJ) beads in a 50:50 mixture in 50 mmol/l Tris buffer, pH 7.0, were added and incubated with the lysates for another 4 hours at 4°C. The immunoprecipitates were washed 4 times in Tris-buffered saline and boiled for 5 minutes in 40 µl of Laemmli buffer containing 0.02% blue bromophenol and 2% beta-mercaptoethanol.

**Immunofluorescence assay**

Cells grown on coverslips were rinsed with PBS and fixed with ice-cold 4% paraformaldehyde for 5 minutes at RT. Subsequently, the cells were blocked with 0.2% Triton X-100 for 30 minutes followed by 5% BSA for 1 hour, washed for 30 minutes, and incubated with primary monoclonal antibodies against BATF2 and p53 overnight at 4°C. The next day, the coverslips were incubated for 1 hour in a dark room with fluorescently conjugated secondary antibody (1:200). Furthermore, the coverslips were stained with DAPI (Vector Laboratories, Burlingame, CA, USA) for 5 minutes at 4°C. Finally, a laser scanning confocal microscope (Leica, Germany) was used to observe the expression in cells.

**Cycloheximide (CHX) chase assay and ubiquitination assay**

A CHX chase assay was used to determine the stability of p53. HGC-27 cells with BATF2 overexpression and control cells were treated with CHX (25 μg/ml) for the indicated times, and the protein levels in the cell lysates were analyzed by western blot. For the ubiquitination assay, cell lysates (an equal amount of protein extracted from stably transfected cells for the ubiquitination analysis) extracted from HGC-27 cells with BATF2 overexpression or knockdown were immunoprecipitated with anti-p53 antibody, and the level of ubiquitinated p53 was assessed with ubiquitin antibody (#3933, Cell Signaling Technology, Danvers, MA).

**References**

1. Kenneth J, Livak TDS. Analysis of relative gene expression data using realtime quantitative PCR and the 2-ΔΔCT method. Methods. 2001;25:402-408.
