# Supplementary material for: m6A modification-mediated BATF2 acts as a tumor suppressor in gastric cancer through inhibition of ERK signaling
Source: Mol Cancer. 2020 Jul 10;19:114. doi: 10.1186/s12943-020-01223-4 (PMC7350710; doi:10.1186/s12943-020-01223-4)
Supplement: Supplementary file 2 — Additional file 2: Table S1. Primers used for qRT-PCR. [file 12943_2020_1223_MOESM2_ESM.docx]

**Table S1.** Primers used for qRT-PCR

| Name | Sequence (5'-3') |
| --- | --- |
| BATF2-F | GCAGGGGTCTTCCTCTAAGC |
| BATF2-R | GCTGCTGAGAGAGCAGGTTT |
| GAPDH-F | TGCACCACCAACTGCTTAGC |
| GAPDH-R | GGCATGGACTGTGGTCATGAG |
