# Supplementary figures and images for: m6A modification-mediated BATF2 acts as a tumor suppressor in gastric cancer through inhibition of ERK signaling
Source: Mol Cancer. 2020 Jul 10;19:114. doi: 10.1186/s12943-020-01223-4 (PMC7350710; doi:10.1186/s12943-020-01223-4)

Figure S1

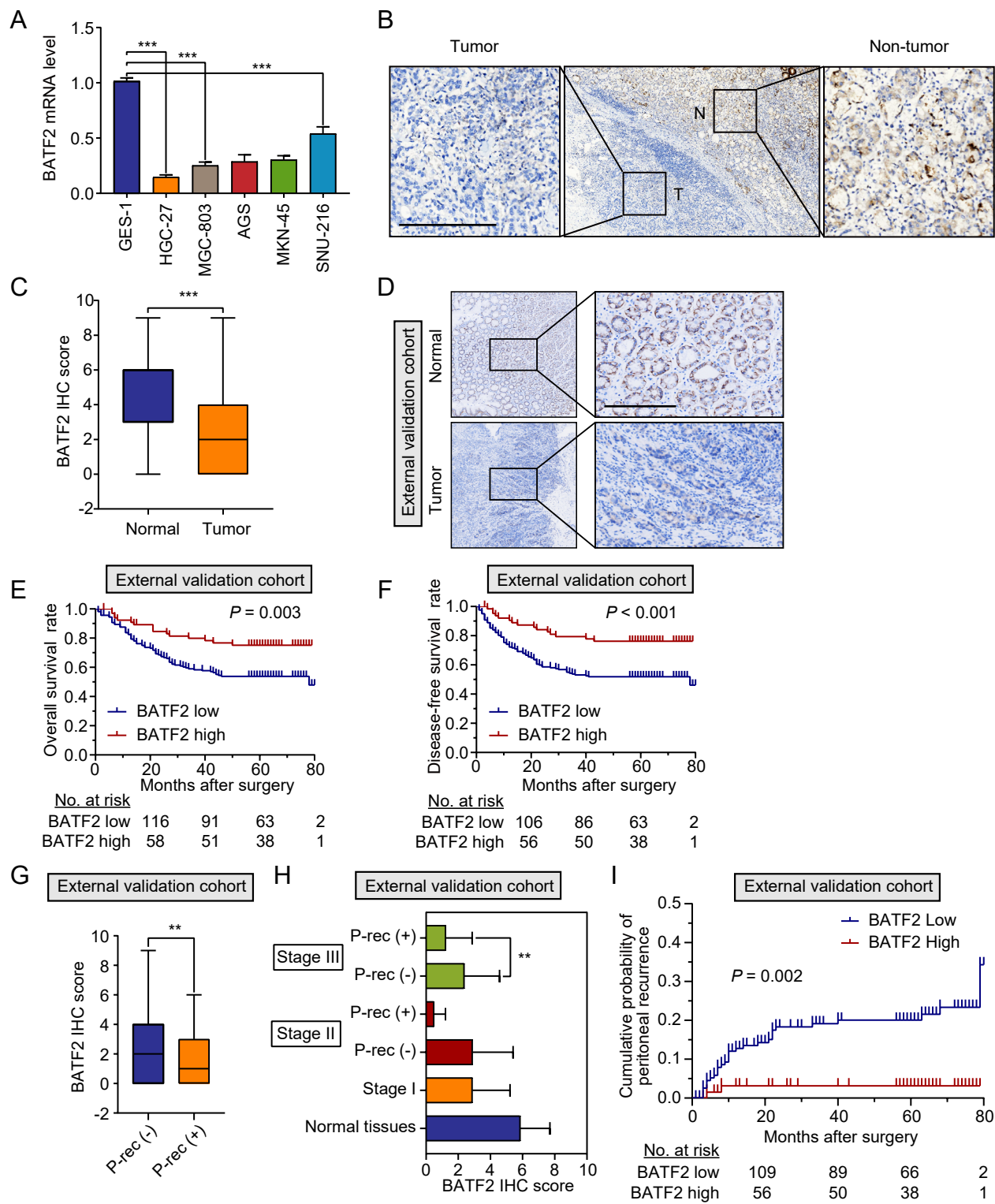

Supplement: Supplementary file 3 — Additional file 3: Figure S1. The expression and prognostic value of BATF2 in GC. a The mRNA levels of BATF2 in human normal gastric epithelial cells (GES-1) and human GC cell lines (HGC-27, MGC-803, AGS, MKN-45 and SNU-216) were detected by real-time PCR and quantified (***P < 0.001). b Representative images of BATF2 IHC staining in gastric tumor and adjacent normal tissues. c For the internal cohort, BATF2 IHC scores in gastric tumor and adjacent normal tissues are shown as box plots (***P < 0.001). d The expression of BATF2 in 232 paraffin-embedded specimens from the external validation cohort was determined by TMA-based IHC staining. Scale bars = 200 μm. e-f Kaplan-Meier analyses of the correlations between BATF2 expression and overall survival or disease-free survival in the external validation cohort. g BATF2 IHC scores in the external validation cohort are shown as box plots. A negative correlation was detected between the BATF2 IHC scores in GC and the frequency of peritoneal recurrence (**P < 0.01; P-rec: peritoneal recurrence). h BATF2 IHC scores in the external validation cohort according to disease stage and the absence or presence of peritoneal recurrence (**P < 0.01; P-rec: peritoneal recurrence). i The cumulative incidence of peritoneal recurrence in GC patients with different BATF2 expression levels from the external validation cohort. [file 12943_2020_1223_MOESM3_ESM.pdf]

**Figure S2**

**A**

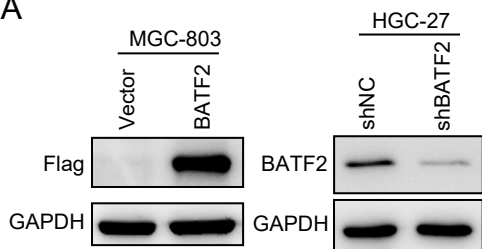

**B**

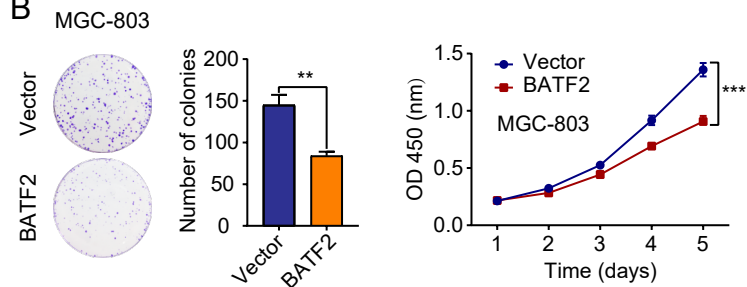

**C**

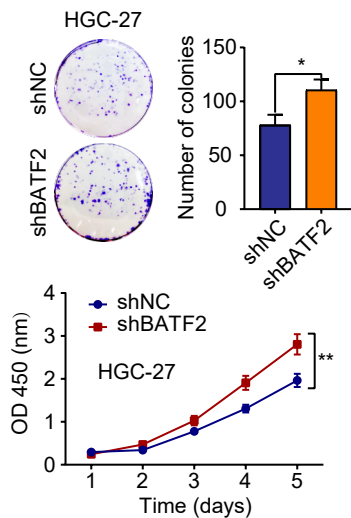

**D**

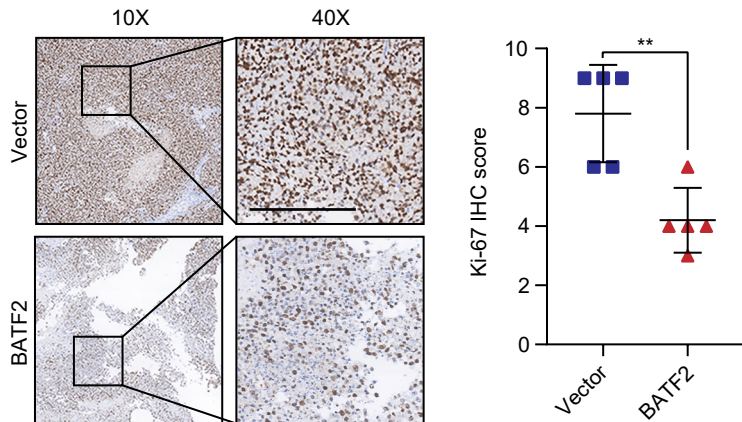

Supplement: Supplementary file 7 — Additional file 7: Figure S2. The effect of BATF2 expression on GC cell proliferation. a MGC-803 and HGC-27 cells with stable BATF2 overexpression or knockdown were created. The changes in BATF2 expression were confirmed using western blotting. b Colony formation and CCK-8 assays with stably transfected MGC-803 cells were performed. Representative images and quantification of the results are presented (**P < 0.01; ***P < 0.001). c Colony formation and CCK-8 assays with stably transfected HGC-27 cells were performed. Representative images and quantification of the results are presented (*P < 0.05; **P < 0.01). d Representative images of Ki-67 IHC staining in xenografted tumors. Scale bars = 200 μm. The Ki-67 IHC scores are shown in the indicated tissues (**P < 0.01). [file 12943_2020_1223_MOESM7_ESM.pdf]

Figure S3

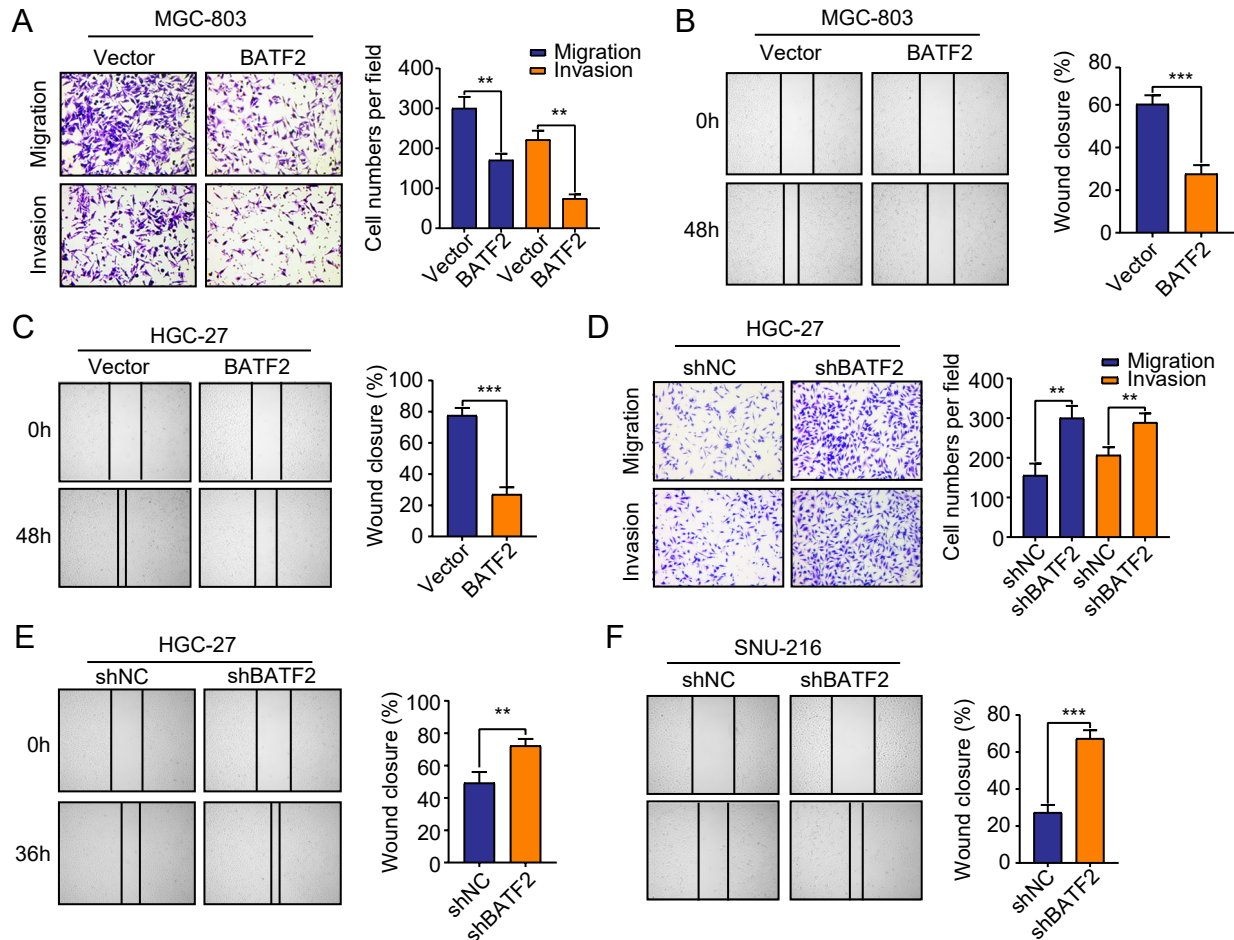

Supplement: Supplementary file 8 — Additional file 8: Figure S3. The effect of BATF2 expression on GC cell invasion and migration. a-b Transwell and wound healing assays with stably transfected MGC-803 cells were performed. Representative images and quantification of the results are presented (**P < 0.01; ***P < 0.001). c-e Transwell and wound healing assays with stably transfected HGC-27 cells were performed. Representative images and quantification of the results are presented (**P < 0.01; ***P < 0.001). f Wound healing assays with stably transfected SNU-216 cells were performed. Representative images and quantification of the results are presented (***P < 0.001). [file 12943_2020_1223_MOESM8_ESM.pdf]

Figure S4

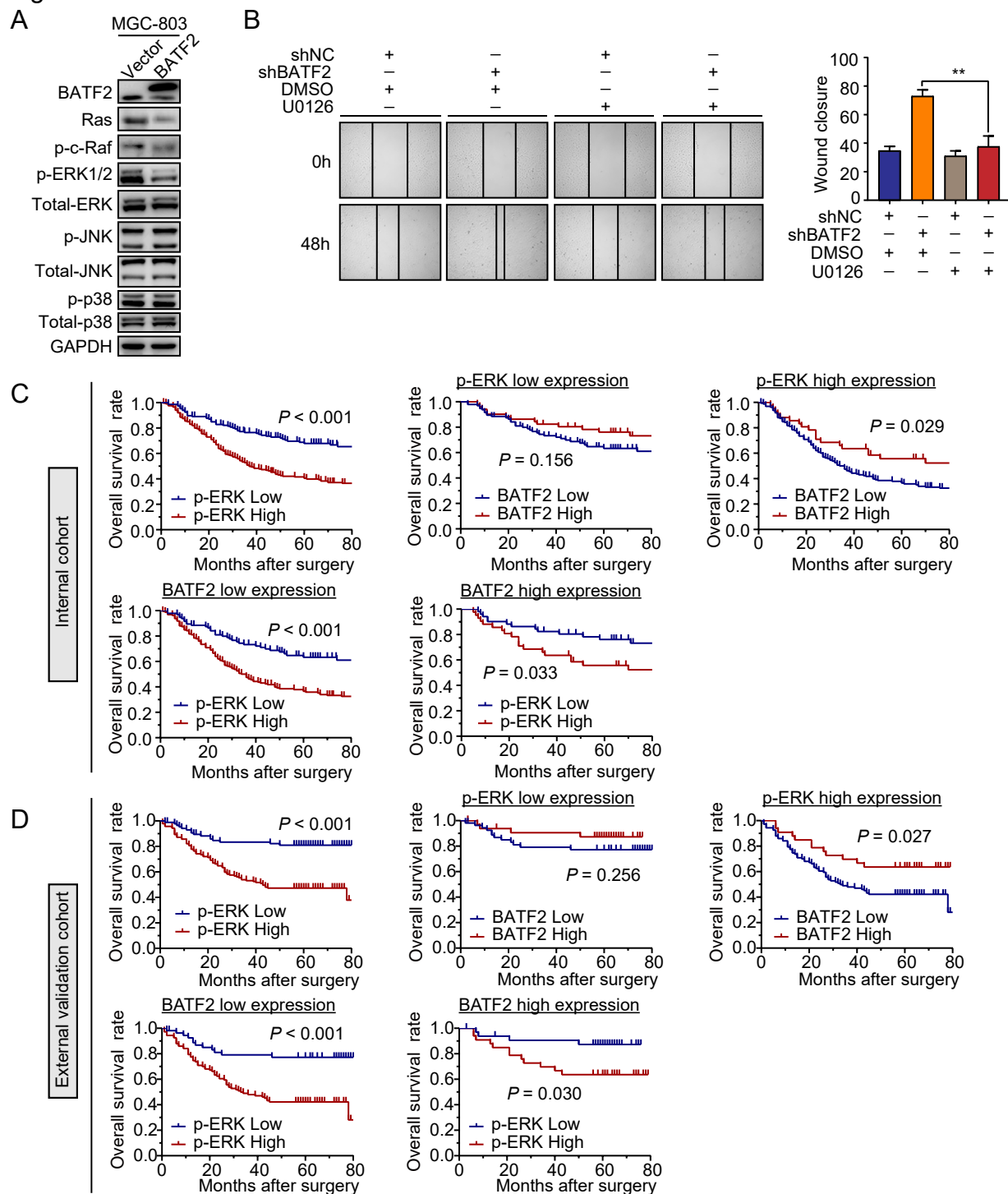

Supplement: Supplementary file 9 — Additional file 9: Figure S4. The clinical value of BATF2 depends on ERK activity. a The expression of critical members of the MAPK pathway in stably transfected MGC-803 cells was examined by western blotting. b Wound healing assays showed that the effect of BATF2 downregulation on SNU-216 cell migration was rescued by U0126 treatment. Representative images are presented (**P < 0.01). c Kaplan-Meier analyses of the correlations between BATF2 and p-ERK expression in the internal cohort. d Kaplan-Meier analyses of the correlations between BATF2 and p-ERK expression in the external validation cohort. [file 12943_2020_1223_MOESM9_ESM.pdf]

# Figure S5

## A

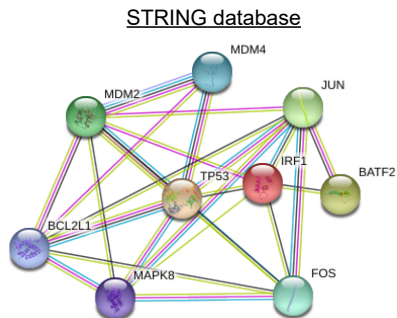

## B

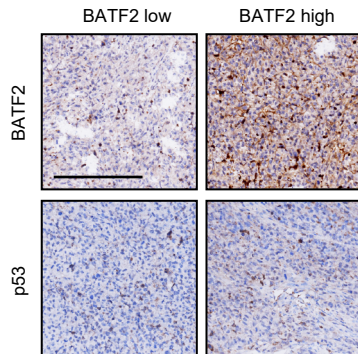

## C

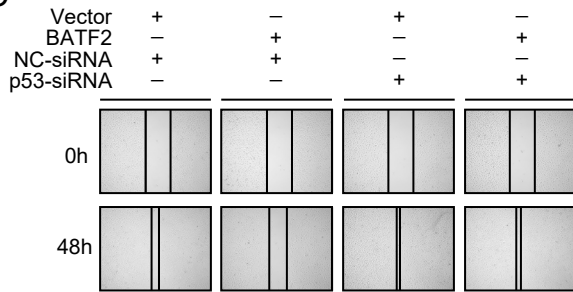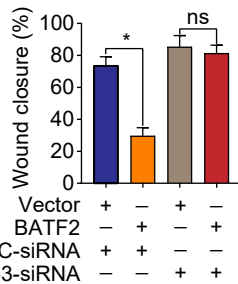

Supplement: Supplementary file 10 — Additional file 10: Figure S5. The relationship between BATF2 and p53. a The online tool STRING was used to predict potential protein-protein interactions. b Representative images of p53 IHC staining in xenograft samples. c Wound healing assays showed that the effect of BATF2 overexpression on HGC-27 cell migration was rescued by p53 siRNA transfection. Representative images are presented (*P < 0.05; ns: no significant difference). [file 12943_2020_1223_MOESM10_ESM.pdf]
