# Supplementary material for: m6A modification-mediated BATF2 acts as a tumor suppressor in gastric cancer through inhibition of ERK signaling
Source: Mol Cancer. 2020 Jul 10;19:114. doi: 10.1186/s12943-020-01223-4 (PMC7350710; doi:10.1186/s12943-020-01223-4)
Supplement: Supplementary file 4 — Additional file 4: Table S2. Univariate and multivariate analyses of overall survival for GC patients [file 12943_2020_1223_MOESM4_ESM.docx]

**Table S2.** Univariate and multivariate analyses of overall survival for GC patients

| Variables | Internal cohort | | | | External validation cohort | | | |
| --- | --- | --- | --- | --- | --- | --- | --- | --- |
|  | Univariate analysis | | Multivariate analysis | | Univariate analysis | | Multivariate analysis | |
|  | HR (95% CI) | *P* | HR (95% CI) | *P* | HR (95% CI) | *P* | HR (95% CI) | *P* |
| Age (≥65 years) | 1.259 (0.933-1.699) | 0.132 |  |  | 1.303 (0.853-1.992) | 0.221 |  |  |
| Sex (Male) | 1.279 (0.893-1.834) | 0.180 |  |  | 0.912 (0.578-1.439) | 0.692 |  |  |
| Tumor size (≥50mm) | 2.460 (1.803-3.356) | <0.001* | 1.723 (1.247-2.381) | 0.001* | 1.335 (0.870-2.046) | 0.186 |  |  |
| Histological grade (Poor) | 1.201 (0.885-1.630) | 0.239 |  |  | 1.738 (1.082-2.794) | 0.022* | 1.200 (0.728-1.980) | 0.475 |
| TNM stage (III&IV) | 3.679 (2.512-5.387) | <0.001* | 2.952 (1.985-4.392) | <0.001* | 3.112 (1.967-4.922) | <0.001* | 2.726 (1.673-4.440) | <0.001* |
| BATF2 (High) | 0.523 (0.356-0.767) | 0.001* | 0.610 (0.415-0.898) | 0.012* | 2.107 (1.226-3.618) | 0.007* | 1.770 (1.026-3.056) | 0.040* |

HR, hazard ratio; CI, confidence interval; **P* < 0.05 was considered significant
