# Supplementary material for: m6A modification-mediated BATF2 acts as a tumor suppressor in gastric cancer through inhibition of ERK signaling
Source: Mol Cancer. 2020 Jul 10;19:114. doi: 10.1186/s12943-020-01223-4 (PMC7350710; doi:10.1186/s12943-020-01223-4)
Supplement: Supplementary file 5 — Additional file 5: Table S3. Analysis of the recurrence sites of GC after curative gastrectomy [file 12943_2020_1223_MOESM5_ESM.docx]

**Table S3.** Analysis of the recurrence sites of GC after curative gastrectomy

| Recurrence patterns | BATF2 expression | | χ^2^ | *P* |
| --- | --- | --- | --- | --- |
|  | Low (n=257) | High (n=95) |  |  |
| Peritoneum |  |  | 6.302 | 0.012* |
| Absent | 226 | 92 |  |  |
| Present | 31 | 3 |  |  |
| Lymph node |  |  | 0.595 | 0.441 |
| Absent | 222 | 85 |  |  |
| Present | 35 | 10 |  |  |
| Hepatic |  |  | 0.857 | 0.354 |
| Absent | 236 | 90 |  |  |
| Present | 21 | 5 |  |  |
| Overall |  |  | 5.379 | 0.020* |
| Absent | 155 | 70 |  |  |
| Present | 102 | 25 |  |  |

**P* < 0.05 was considered significant
