# Supplementary material for: m6A modification-mediated BATF2 acts as a tumor suppressor in gastric cancer through inhibition of ERK signaling
Source: Mol Cancer. 2020 Jul 10;19:114. doi: 10.1186/s12943-020-01223-4 (PMC7350710; doi:10.1186/s12943-020-01223-4)
Supplement: Supplementary file 6 — Additional file 6: Table S4. Univariate and multivariate analyses of peritoneal recurrence after gastrectomy [file 12943_2020_1223_MOESM6_ESM.docx]

**Table S4.** Univariate and multivariate analyses of peritoneal recurrence after gastrectomy

| Variables | Internal cohort | | | | External validation cohort | | | |
| --- | --- | --- | --- | --- | --- | --- | --- | --- |
|  | Univariate analysis | | Multivariable analysis | | Univariate analysis | | Multivariable analysis | |
|  | HR (95% CI) | *P* | HR (95% CI) | *P* | HR (95% CI) | *P* | HR (95% CI) | *P* |
| Age (≥65 years) | 1.077 (0.543-2.133) | 0.832 |  |  | 0.861 (0.411-1.801) | 0.690 |  |  |
| Sex (Male) | 0.638 (0.315-1.289) | 0.210 |  |  | 0.537 (0.271-1.064) | 0.075 |  |  |
| Tumor size (≥50 mm) | 4.211 (1.957-9.060) | <0.001* | 2.519 (1.153-5.506) | 0.021* | 1.373 (0.683-2.761) | 0.374 |  |  |
| Histological grade (Poor) | 4.173 (1.617-10.766) | 0.003* | 3.196 (1.231-8.154) | 0.017* | 1.975 (0.891-4.379) | 0.094 |  |  |
| TNM stage (III&IV) | 9.383 (2.857-30.819) | <0.001* | 5.729 (1.691-19.414) | 0.005* | 4.942 (2.142-11.404) | <0.001* | 4.224 (1.825-9.774) | 0.001* |
| BATF2 (High) | 0.225 (0.069-0.738) | 0.014* | 0.301 (0.092-0.986) | 0.047* | 7.206 (1.725-30.096) | 0.007* | 5.741 (1.368-24.087) | 0.017* |

HR, hazard ratio; CI, confidence interval; **P* < 0.05 was considered significant
