# Supplementary material for: m6A modification-mediated BATF2 acts as a tumor suppressor in gastric cancer through inhibition of ERK signaling
Source: Mol Cancer. 2020 Jul 10;19:114. doi: 10.1186/s12943-020-01223-4 (PMC7350710; doi:10.1186/s12943-020-01223-4)
Supplement: Supplementary file 11 — Additional file 11: Figure S6. Relative expression of BATF2 and METTL3 in GC shown by H. pylori status. a BATF2 and METTL3 IHC scores from the internal cohort. b Relative expression of BATF2 and METTL3 from the ACRG dataset (GSE62254). ns: no significant difference. [file 12943_2020_1223_MOESM11_ESM.pdf]

Figure S6

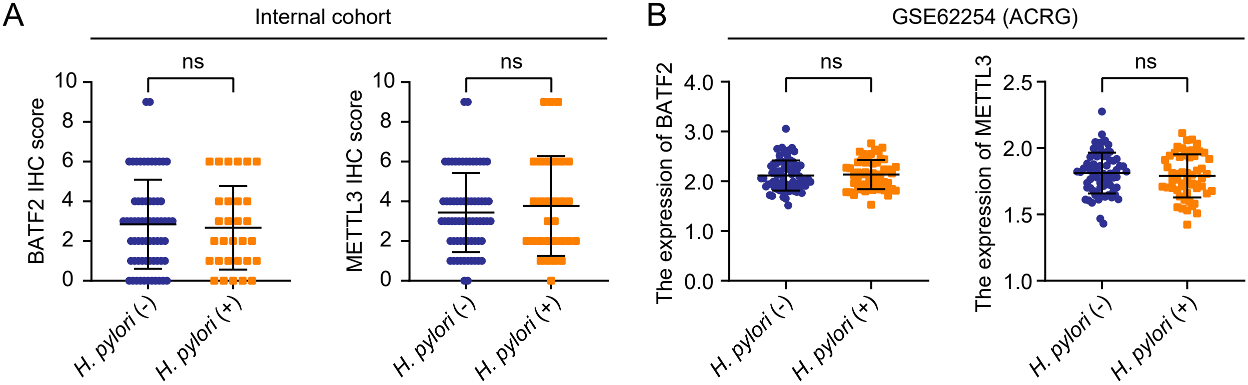
A
Internal cohort
ns
ns
BATF2 IHC score
METTL3 IHC score
10
8
6
4
2
0
H. pylori (-)
H. pylori (+)
B
GSE62254 (ACRG)
ns
ns
The expression of BATF2
The expression of METTL3
4.0
3.0
2.0
1.0
0.0
2.5
2.0
1.5
1.0
